# Association between immune cells and allergic purpura: a Mendelian randomization study

**DOI:** 10.1186/s13052-025-01847-6

**Published:** 2025-04-10

**Authors:** Wei Xian, Huiyi Zhang, Huasong Zeng

**Affiliations:** 1 Department of Pediatric Allergy, Immunology and Rheumatology, Guangzhou Women and Children’s Medical Center, Guangzhou Medical University, Guangzhou, Guangdong Province China; 2 Sun Yat-Sen University School of Medicine, Shenzhen Campus of Sun Yat-sen University, No. 66, Gongchang Road, Guangming District, Shenzhen, 518107 Guangdong Province China

**Keywords:** Allergic purpura, Immune cells, Mendelian randomization, Single nucleotide polymorphism

## Abstract

**Background:**

Increasing evidence indicates a substantial correlation between the immune cells and the risk of allergic purpura. We utilized Mendelian randomization (MR) to investigate causal effect of immune cell on allergic purpura.

**Methods:**

Genetic instrumental variables for immune cells were sourced from an extensive genome-wide association study (GWAS) comprising 3757 participants. Summary statistics of allergic purpura, involving 470 cases and 216,099 controls, were obtained from FinnGen. The primary analysis employed the inverse-variance weighted (IVW) method. Rigorous sensitivity analyses including MR-Egger, weighted median and MR-PRESSO were conducted to ensure the reliability of the causal estimate.

**Results:**

We identified two immunophenotypes associated with an increased risk of allergic purpura: HLA-DR on CD14 + CD16- monocyte (OR: 1.2379; 95% CI: 1.0612–1.4440; *P* = 0.0066) and CD11b on basophil (OR: 1.2973; 95% CI: 1.0905–1.5433; *P* = 0.0033). The sensitivity analyses consistently yielded similar results for these immunophenotypes.

**Conclusions:**

Our analyses confirmed a potential causal effect of HLA-DR on CD14 + CD16- monocyte, as well as CD11b on basophils, in relation to the risk of allergic purpura. Further studies are necessary to clarify the mechanisms by which these immunophenotypes influence the development of allergic purpura.

**Supplementary Information:**

The online version contains supplementary material available at 10.1186/s13052-025-01847-6.

## Introduction

Allergic purpura, also known as IgA vasculitis, is an immune complex vasculitis primarily affecting small vessels [1] and leads to palpable purpura, arthralgia/arthritis, bowel angina, and haematuria/proteinuria [2, 3]. Allergic purpura is the most common childhood systemic vasculitis, with an estimated incidence of 20.4 per 100,000 children [2]. The major morbidity of allergic purpura is kidney involvement, known as IgA vasculitis nephritis (IgAV-N), which can develop within 4–12 weeks after disease onset [4]. Early detection of allergic purpura is thus very important.

Currently, the pathogenic mechanisms of IgA vasculitis (IgAV) are not fully understood. Multiple studies have indicated that genetic factors, infections, and immune dysregulation play significant roles in disease progression. For instance, there is often a history of respiratory infections or vaccinations prior to the onset of IgAV [1, 5]. Certain human leukocyte antigen (HLA) alleles, such as HLA-B35 and HLA-DRB1*01, are associated with susceptibility to IgAV [6, 7]. Additionally, increasing research highlights the critical role of immune dysregulation in the disease course of IgAV. The pathophysiology of allergic purpura likely involves dysregulation of the immune system initiating an inflammatory response and injury. Immune complexes produced by B cells deposit in the glomeruli of the kidney [8, 9], while monocytes and T cells accumulate, leading to damage [10]. Previous studies have shown that the activation levels of CTL and NK cells are increased in allergic purpura [11]. Additionally, the frequency of Tr1 cells in peripheral blood is reduced in IgAV patients, which may be associated with increased EGR2 expression [12]. Neutrophils are also believed to be involved in the pathogenesis of IgAV [13].

Renal involvement is an important factor in determining the prognosis of IgAV. Up to 20-80% of children with IgAV exhibit symptoms of nephritis, and 1–7% of children diagnosed with IgA vasculitis nephritis (IgAV-N) are at risk of progressing to renal failure or end-stage renal disease [14]. The excessive differentiation of Th2 cells, Th17 cells, and Tfh cells may play a role in the onset and progression of IgAV-N [15]. This suggests that the immune system’s response, characterized by the overproduction of these specific cell types, could be a contributing factor to pathogenesis of IgAV-N. Therefore, the relationship between immune cells and IgAV is highly complex and closely intertwined, and it is recommended to investigate causal effect of immune cell on IgAV.

However, past observational studies on the relationship between immune cells and allergic purpura have been affected by confounding factors. Mendelian randomization (MR) has emerged as a novel method to explore the causal relationship between immune cells and allergic purpura [16]. MR utilizes genetic variants as instrumental variables, enabling the evaluation of the relationship between exposure and outcome while mitigating confounding factors [17]. Thus employing a two-sample Mendelian randomization (MR) analysis approach with data from genome-wide association studies (GWAS) overcomes the limitations of observational studies and enhances the study’s methodological rigor. This research not only gives us a deeper comprehension of the complex interplay between immune cells and allergic purpura, but also paves the way for targeted interventions and therapeutic strategies.

## Methods

We used two-sample MR to investigate causal association between immune phenotypes and allergic purpura. Only publicly available GWAS summary data were included in our study. Therefore, no additional informed consent or ethical review was required for our use of these data. We reported our study based on STROBE-MR guideline and STREGA guideline [18, 19].

### Genome-wide association study (GWAS) data sources for allergic purpura

The genetic summary statistics underpinning research on allergic purpura originated from a genome-wide association study (GWAS) involving 470 cases and 216,099 controls with European ancestry from FinnGen R5 release [20]. The FinnGen project is an ongoing project and R5 dataset was released to public in May 11th 2021, and we obtained the data in October 5th 2023 [21]. Cases were selected on the basis of International Classification of Diseases, 10th version (ICD10) ICD-10: D690, ICD-8: 2870. DNA specimens of FinnGen were collected from a consortium of participating Finnish biobanks. The extraction process involved either whole blood or buffy coat, adhering to each biobank’s standardized protocols and then dispatched to the centralized logistics facility at the the Biobank of the Finnish Institute for Health and Welfare (THL Biobank). THL Biobank laboratory meticulously aliquoted and normalized the samples into 96-well plates. Subsequently, these prepared plates were devivered to the Affymetrix Research Services Laboratory at Thermo Fisher Scientific for further analysis. FinnGen individuals were genotyped with Illumina and Affymetrix chip arrays and imputed using the population-specific SISu v3 imputation reference panel of 3,775 whole genomes [20]. Summary data were adjusted for confounding factors like age, sex, technical covariates, and genetic principal components.

**Fig. 1:**
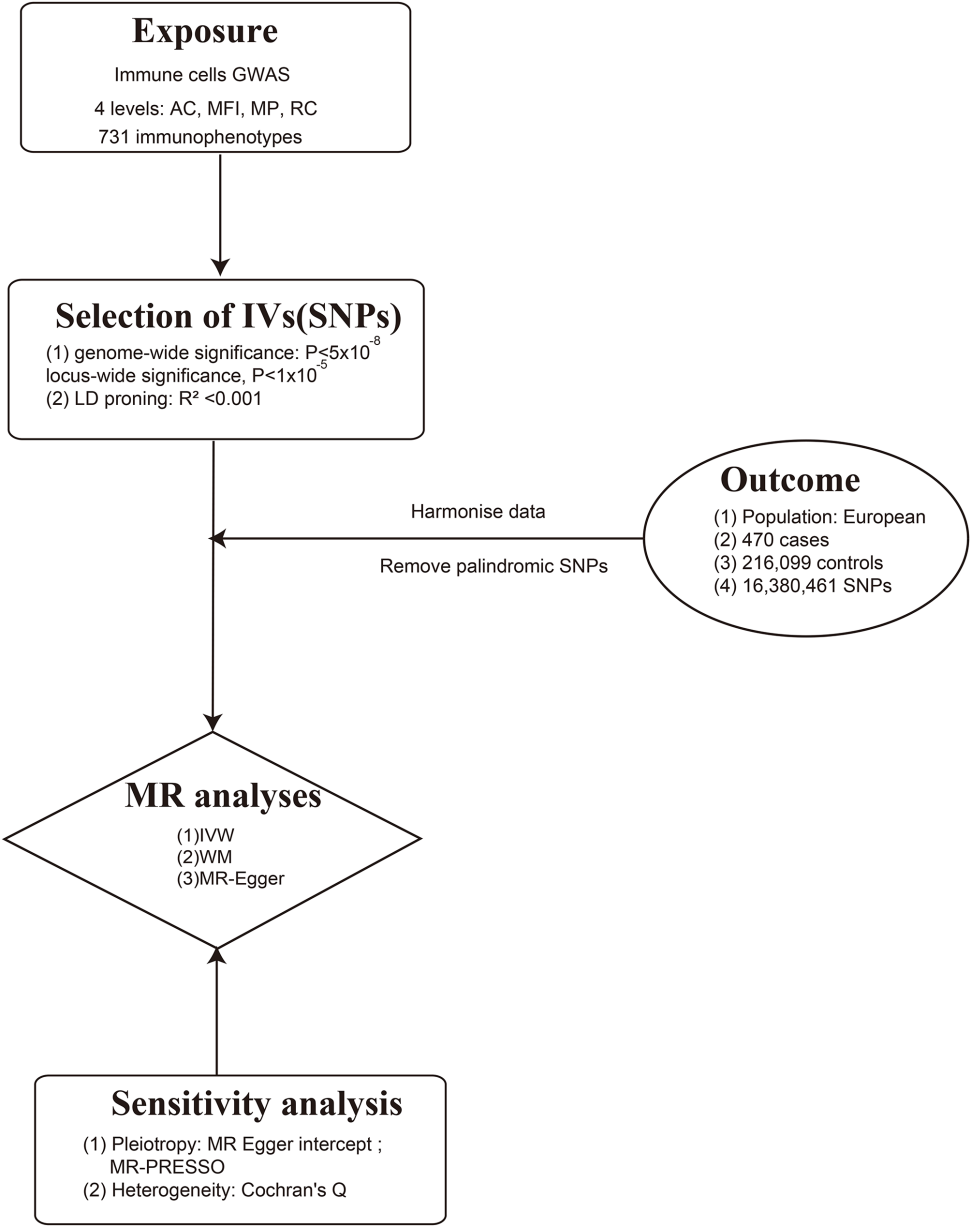
Mendelian randomization flowchart for investigating causal relationship between immune cells and allergic purpura

SNPs associated with immune phenotypes were extracted from with GWAS summary statistics conveyed by Orru V et al. [22]. GWAS summary data of immunophenotypes was released to GWAS catalog in Sep 14th 2020 and we obtained the data in Oct 5th 2023 [22]. The original study included 3757 European individuals. Peripheral blood was collected in heparin tube. Cell phenotyping was conducted at the same recruitment center, using erythrocyte-lysed fresh samples, within two hours following blood collection. This comprehensive study profiled 731 immunophenotypes by flow cytometry, covering a spectrum of features like absolute cell counts by BD TruCount absolute counting tubes, median fluorescence intensities calculating by the Spearman coefficient, morphological parameters assessing by light scattering measured using two optical detectors, and relative cell counts which are ratios between cell levels. Samples were stained with 7 antibody panels, including TBNK panel, Treg panel, maturation stages of T-cell panel, DC panel, B-cell panel, monocyte panel, myeloid cell panel [22]. The genotyping process incorporated approximately 22 million SNPs, utilizing four different Illumina arrays: OmniExpress, ImmunoChip, Cardio-MetaboChip, and ExomeChip and imputation with a Sardinian sequence-based reference panel. Data was adjusted for covariates like sex, age, and age squared. Figure 1 illustrates the design of our study for investigating potential causal links between immune cells and allergic purpura. Notably, the use of publicly available summary data obviated the need for additional ethical approvals or participant consents. Details of data source were shown in Table 1.

**Table 1:** Details of the datasets used in the analyses

Phenotype	Sample size	Population	Data source	PMID
**Immune phenotypes**	3757	European	https://www.ebi.ac.uk/gwas/downloads/summary-statistics	32,929,287
**Allergic purpura**	216,569	European	https://gwas.mrcieu.ac.uk/datasets/finn-b-D3_ALLERGPURPURA/	36,653,562

### Instrumental variables (IVs)

**Fig. 2:**
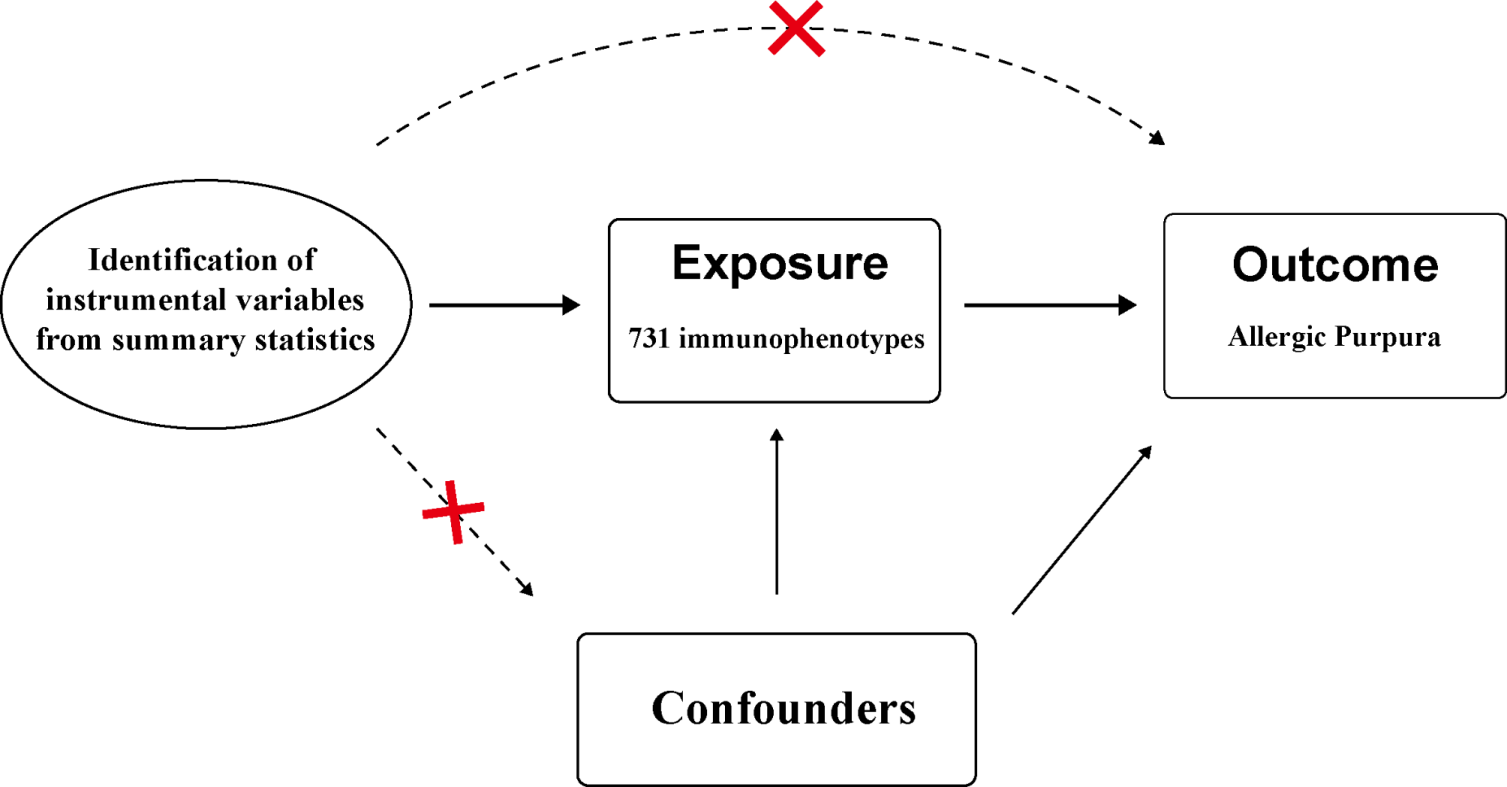
Assumptions in MR studies: a brief overview

Mendelian Randomization (MR) relies on three fundamental assumptions to ensure the validity of causal inference. Firstly, the genetic variant used as an instrumental variable (IV) should be strongly associated with the exposure of interest. Secondly, genetic variants were not associated with confounders. Lastly, the instrumental variables influences the outcome solely through its impact on the exposure. These assumptions constitute the bedrock of MR analysis, employing genetic variants as tools to deduce causal connections between exposures and outcomes, as illustrated in Fig. 2 [19].

The selection of instrumental variables (IVs) for investigating causal association between immune phenotypes and allergic purpura follows a rigorous process to ensure robust results. When extracting IVs associated with immune phenotypes, a locus-wide *p*-value threshold of *p* < 1.0 × 10^− 5^ is applied. Independence among IVs is maintained through LD threshold (R^2^ < 0.001) and a clumping distance of 10,000 kb, utilizing the “TwoSampleMR” package with 1000 Genomes EUR data. Instrument strength of IV is assessed using F-statistics(β^2^/se^2^), with values exceeding 10 indicate minimal weak instrumental bias [23]. SNPs featuring *A*/*T* or *G*/*C* alleles, termed palindromic SNPs, introduce ambiguity into the identity of the effect allele in the exposure and outcome GWASs. To maintain reference strand integrity, palindromic SNPs with effect allele frequencies between 0.3 and 0.7 are excluded. When a particular SNP is absent from the outcome dataset, linkage disequilibrium proxy SNPs can be used. We set the threshold of r^2^ at 0.8 to ensure that the proxy SNP and target SNP have a strong correlation so that the proxy SNP can replace the target SNP as an IV [24].

### Statistical analysis

In investigating the causal connection between immune cells and allergic purpura, we employed several methods including inverse variance weighted (IVW), MR-Egger regression, and weighted median method. IVW method is the main analysis of our study, which assumes the absence of horizontal pleiotropy [25]. The random-effect IVW method, utilizing a meta-analysis approach, combines Wald ratio from each single nucleotide polymorphism (SNP) to generate an overall causal effect estimate for the impact of immune cells on allergic purpura adjusting heterogeneity among SNPs. The weighted median method offers a reliable estimate in MR analyses, even when up to 50% of the instrumental variables used are invalid [26].

Sensitivity analysis can make sure the robustness of causal association between exposures and outcomes. MR-Egger regression and MR-PRESSO were used to evaluate potential horizontal pleiotropy. MR-Egger is a method developed for two-sample MR settings that combines Wald ratio (or ratio estimates) together into a meta-regression (with an intercept and slope parameter) to estimate the causal effect adjusted for any directional pleiotropy [27]. MR-PRESSO is an extension to the inverse variance weighted (IVW) method, which attempts to perform the same form of outlier removal as in the generalized summary MR (GSMR) method and Radial MR [28]. If the *p*-value of the MR-Egger intercept was below 0.05 or *p*-value of the MR-PRESSO global test was less than 0.05, it implied the existence of horizontal pleiotropy in the SNPs [27, 28]. Assessment of heterogeneity among SNPs was conducted using Cochran’s Q test [29]. The TwoSampleMR package (version 0.5.7) in R (version 4.3.0) was the analysis tool of choice.

## Results

### Main results of the immune cell traits with the risk of allergic purpura

**Fig. 3:**
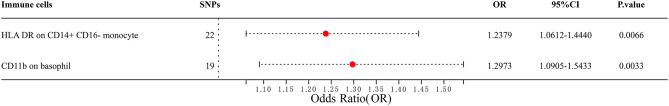
Associations of genetically determined immune cell traits with allergic purpura risk

F-statistics, ranging from 19.55 to 2381.77 were computed for 731 immunophenotypes, all exceeding 10, indicating minimal weak instrument bias. Detailed information on SNPs for each trait is available in Supplementary Table (1). MR results for all traits and their association with allergic purpura risk are outlined in Supplementary Table 3, revealing two traits with suggestive associations (*p* < 0.01) using the IVW method, as depicted in Fig. 3. Instrumental variables for these traits are provided in Supplementary Tables 1 and 2. We analyzed causal effect of 731 immunophenotypes on allergic purpura. While 729 immunophenotypes exhibited no significant correlation with allergic purpura, we found that HLA-DR on CD14 + CD16- monocyte in monocyte panel and CD11b on basophil in myeloid cell panel had significant effect on risk of developing allergic purpura, respectively. None of the immunophenotypes in TBNK panel, Treg panel, maturation stages of T cells panel, DC panel showed significant effect on developing allergic purpura according to results of IVW method. Results were illustrated in Fig. 3 and detailed in Supplementary Table 4.

Using the IVW method, higher HLA-DR on CD14 + CD16- monocyte was revealed to lead to a higher risk of getting allergic purpura using the IVW method (OR: 1.2379; 95% CI: 1.0612–1.4440; *P* = 0.0066), and weighted median analyses showed similar result (OR: 1.3511; 95% CI: 1.0939–1.6687; *P* = 0.0052). The results of the MR Egger (OR: 1.2863; 95% CI: 0.9640–1.7165; *P* = 0.1026) and weighted median (OR: 1.3511; 95% CI: 1.0939–1.6687; *P* = 0.0052) analyses are consistent with IVW. Both MR-PRESSO(*P* = 0.4780) and MR-Egger intercept (*P* = 0.7594) analyses were > 0.05, detected no outliers or directional pleiotropic effects. Meanwhile, CD11b on basophil is suggested to be one of the risks of developing allergic purpura using the IVW method (OR: 1.2973; 95% CI: 1.0905–1.5433; *P* = 0.0033). The results of the weighted median (OR:1.1712; 95% CI: 0.9220–1.4877; *P* = 0.1954) analyses are consistent with IVW. Similarly, both MR-PRESSO (*P* = 0.408) and MR-Egger intercept(*P* = 0.0691) analyses were > 0.05, detected no outliers or directional pleiotropic effects. The *p*-value the Cochran’s Q of both HLA-DR on CD14 + CD16- monocyte (*P* = 0.4770) and CD11b on basophil(*P* = 0.3455) were both greater than 0.05, indicating no significant heterogeneity among SNPs associated with these two immunophenotypes. Result of MR-Egger intercept and Cochran’s Q of all immunophenotype are presented in supplementary Tables 5 and 6.

## Discussion

Based on large publicly available genetic data, we explored causal associations between 731 immune cell traits and allergic purpura. In this study, two immunophenotypes, HLA-DR on CD14 + CD16- monocyte and CD11b on basophil, was found to have causal effects on allergic purpura.

Our study found that the risk of allergic purpura increased with an increase in HLA-DR on CD14 + CD16- monocyte. Previous studies have shown that the number of monocytes in the circulating blood of patients with allergic purpura is significantly higher than that of the control group without the disease [30]. This may be due to the important role played by monocytes in promoting inflammation and anti-inflammatory processes at inflammatory sites [31].

CD14 + CD16- was referred to as ‘‘classical’’ monocytes, making up 80–90% of the monocyte pool [31]. Classical monocytes have pro-inflammatory activities, antigen-presentation, tissue remodeling and anti-inflammatory abilities. Human monocytes can be identified by their HLA-DR expression [32]. HLA-DR is a class II MHC molecule that is involved in antigen presentation by monocytes. When stimulated with staphylococcal enterotoxin B, CD14 + CD16- monocytes show increased expression of HLA-DR, leading to enhanced ability to activate the proliferation of CD4 + T cell [33]. It has been reported that Th2 cells, Th17 cells, and Tfh cells may play a role in the onset and progression of IgAV-N [15]. Previous research has also pointed out that HLA class II genes have been implicated in susceptibility to IgAV, with special emphasis on HLA-DRB1 alleles [34, 35].

The increase of CD11b on basophil also leads to an increased risk of allergic purpura. The basophil is well-known for its role in allergic inflammation and it also has immunoregulatory functions in both innate and adaptive immunity. Previous studies have shown that basophils can be found in perivascular areas in patients with allergic purpura, and compared to the recruitment of eosinophils, there is a predominance of basophils [36].CD11b belongs to the integrin family β2 group, and when combined with CD18, it forms CR3, which is involved in immune cell adhesion, inflammation, and phagocytosis. Crosslinking of CD11b with other adhesion molecules can promote activation of basophil which will participate in inflammatory reactions [37]. Increased level of CD11b may indicate that basophils are more easily activated, leading to the higher occurrence of allergic purpura.

This study conducted a two-sample Mendelian randomization (MR) analysis based on published large-scale GWAS cohorts, with a sample size of 216,569 individuals, ensuring high statistical efficiency. The conclusions drawn in this study are reliable as they are based on genetic instrumental variables and employ various causal inference methods using MR. These results are not influenced by horizontal pleiotropy or other factors. The research findings innovatively demonstrate that elevated levels of HLA-DR on CD14 + CD16- monocytes and CD11b on basophils are associated with the occurrence of allergic purpura. However, there are limitations to consider in this study. First, a predominant proportion of participants in the GWAS were European descent so the generalizability of the study’s outcomes to other racial or ethnic groups may be compromised. Secondly, this study didn’t explore the diverse subtypes or specific characteristics of allergic purpura. Thirdly, our study didn’t include expression data of HLA-DR on CD14 + CD16- monocyte and CD11b on basophil in allergic purpura patients and healthy people. In summary, it can be seen that a call for further research is warranted. Future investigations should encompass larger and more diverse populations, incorporate a consideration of cell function, prioritize replication in independent cohorts, and explore associations with specific allergic purpura subtypes. Further research into the underlying mechanisms should be conducted to clarify the pathophysiological pathways of immune cells implicated in the onset and renal manifestations of IgAV. Addressing these aspects will undoubtedly contribute to a more nuanced and enriched understanding of the intricate relationship between immune cells and allergic purpura.

## Conclusions

In conclusion, we have demonstrated the causal effect of HLA-DR on CD14 + CD16- monocyte and CD11b on basophil and allergic purpura through a comprehensive MR analysis, highlighting the complex pattern of interactions between the immune system and allergic purpura.

Furthermore, our research significantly reduced the impact of unavoidable confounding factors and other factors. It may provide a new avenue for researchers to explore the biological mechanisms of IgAV and can lead to exploration of earlier intervention and treatment.

These promising findings could provide new targets for IgAV treatment, guiding the development of targeted therapies and reducing the incidence of IgAV sequelae.

## Electronic supplementary material

Below is the link to the electronic supplementary material.

Supplementary Material 1

Supplementary Material 2

Supplementary Material 3

Supplementary Material 4

Supplementary Material 5

Supplementary Material 6

Supplementary Material 7

## Data Availability

GWAS summary statistics of immune phenotypes can be obtained from the GWAS Catalog with accession numbers from GCST90001391 (https://www.ebi.ac.uk/gwas/studies/GCST90001391) to GCST90002121 (https://www.ebi.ac.uk/gwas/studies/GCST90002121). GWAS summary statistics of allergic purpura can be obtained from MRC-IEU database(https://gwas.mrcieu.ac.uk/datasets/finn-b-D3_ALLERGPURPURA/) or from FinnGen database (https://finngen.gitbook.io/documentation/r5/data-download).

## Abbreviations

CI: Confidence interval
EGR2: Early growth response 2
GWAS: Genome-wide association study
GSMR: Generalized summary MR
HLA-DR: Human leukocyte antigen – dr isotype
IgA: Immunoglobulin A
IgAV: Immunoglobulin A Vasculitis
IgAV-N: IgA vasculitis nephritis
IVs: Instrumental variables
IV: Instrumental variable
IVW: Inverse-variance weighted
MHC: Major histocompatibility complex
MR: Mendelian randomization
OR: Odds ratio
SNP: Single nucleotide polymorphism
