# Supplementary material for: Association between immune cells and allergic purpura: a Mendelian randomization study
Source: Ital J Pediatr. 2025 Apr 10;51:112. doi: 10.1186/s13052-025-01847-6 (PMC11987331; doi:10.1186/s13052-025-01847-6)
Supplement: Supplementary file 1 — Supplementary Material 1 [file 13052_2025_1847_MOESM1_ESM.docx]

**Supplementary Information**

**Title**

Association between immune cells and allergic purpura: a Mendelian randomization study

**Authors and affiliations**

Wei Xian^1†^, Huiyi Zhang^2†^, Huasong Zeng^1*^

^1^ Department of Pediatric Allergy, Immunology and Rheumatology, Guangzhou Women and Children’s Medical Center, Guangzhou Medical University, Guangzhou, Guangdong Province, China.

^2^ Sun Yat-Sen University School of Medicine, Shenzhen Campus of Sun Yat-sen University, No. 66, Gongchang Road, Guangming District, Shenzhen, Guangdong Province, 518107, China.

^†^These authors contributed equally to this work and share first authorship.

**Corresponding author**

Huasong Zeng, Department of Pediatric Allergy, Immunology and Rheumatology, Guangzhou Women and Children’s Medical Center, Guangzhou Medical University, Guangzhou, Guangdong Province, China. E-mail: zenghuasong@gwcmc.org. ORCID: 0000-0002-7166-0941

**Catalogue of supplementary information**

**Supplementary Table 1**: Descriptive detail of SNP associated with immune cells.

**Supplementary Table 2**: Detailed information of instrumental variables used in MR analyses about immune cells on allergic purpura.

**Supplementary Table 3**: MR analysis of all immune cells on allergic purpura.

**Supplementary Table 4**: Effect estimates of the associations of allergic purpura in the MR analyses.

**Supplementary Table 5**: Directional horizontal pleiotropy assessed by intercept term in MR Egger regression of the association between immune cells and allergic purpura.

**Supplementary Table 6**: The heterogeneity of immune cells instrumental variables.
